# Imaging through diffuse media using multi-mode vortex beams and deep learning

**DOI:** 10.1038/s41598-022-05358-w

**Published:** 2022-01-28

**Authors:** Ganesh M. Balasubramaniam, Netanel Biton, Shlomi Arnon

**Affiliations:** Department of Electrical and Computer Engineering, Ben-Gurion University of the Negev, 8441405 Beersheba, Israel

**Keywords:** Optics and photonics, Applied optics, Optical physics

## Abstract

Optical imaging through diffuse media is a challenging issue and has attracted applications in many fields such as biomedical imaging, non-destructive testing, and computer-assisted surgery. However, light interaction with diffuse media leads to multiple scattering of the photons in the angular and spatial domain, severely degrading the image reconstruction process. In this article, a novel method to image through diffuse media using multiple modes of vortex beams and a new deep learning network named “LGDiffNet” is derived. A proof-of-concept numerical simulation is conducted using this method, and the results are experimentally verified. In this technique, the multiple modes of Gaussian and Laguerre-Gaussian beams illuminate the displayed digits dataset number, and the beams are then propagated through the diffuser before being captured on the beam profiler. Furthermore, we investigated whether imaging through diffuse media using multiple modes of vortex beams instead of Gaussian beams improves the imaging system's imaging capability and enhances the network's reconstruction ability. Our results show that illuminating the diffuser using vortex beams and employing the “LGDiffNet” network provides enhanced image reconstruction compared to existing modalities. When employing vortex beams for image reconstruction, the best NPCC is − 0.9850. However, when using Gaussian beams for imaging acquisition, the best NPCC is − 0.9837. An enhancement of 0.62 dB, in terms of PSNR, is achieved using this method when a highly scattering diffuser of grit 220 and width 2 mm (7.11 times the mean free path) is used. No additional optimizations or reference beams were used in the imaging system, revealing the robustness of the “LGDiffNet” network and the adaptability of the imaging system for practical applications in medical imaging.

## Introduction

The interaction of light with matter is often characterized by manifold scattering and absorption events. The effects of light scattering in media manifest as low visibility during foggy conditions, blurring in images, and loss of information in medical imaging, to name a few examples. The amount of scattering in any given medium depends on the light source structure, intensity and wavelength, and the medium's optical properties. The amount of scattering can be measured. These measurements offer deep insight into the properties of the medium and the behavior of light propagating through it. Such studies have led to optical detection using visible and infrared light, which has become hugely popular in biomedicine due to its robustness and tremendous advances in computer science^1–9^. Compared to other classical biomedical techniques like X-ray imaging^10^, optical imaging using visible and near-infrared (NIR) light is non-ionizing. It causes no harm to the samples during screening. It is also cheaper to implement than conventional biomedical imaging techniques such as magnetic resonance imaging (MRI)^11^. However, optical imaging systems that use visible and NIR light suffer from optical blurring and photon noise phenomena^12–14^. Primarily caused by the interaction of light with tissue and their subsequent propagation, these effects often contribute to the image's degradation, affecting its resolution and the characterization of edges^15^. Absorption and multiple scattering in all directions also lead to loss of information in light that does not reach the beam profiler's aperture^16^. The reconstruction of displayed objects in tissues from the images obtained by the beam profiler is also made difficult, time-consuming, and cumbersome due to the complex mathematical models involved in image reconstruction calculations^17,18^. Hence, an optical imaging system needs to retrieve complete information about the displayed object, improve resolution, and reduce the complexity of analytical models to reconstruct the displayed object. The former two objectives can be achieved using structured light sources, and the latter can be addressed by implementing deep learning algorithms with sufficient training. Studies have shown that imparting a topological charge to beams used for optical imaging enhances optical imaging systems^19–22^ and improves the beams' penetration depth^5,23–25^. It has also been demonstrated that vortex beams have more transmissivity through scattering media than Gaussian beams^22,23,25,26^.

**Figure 1:**
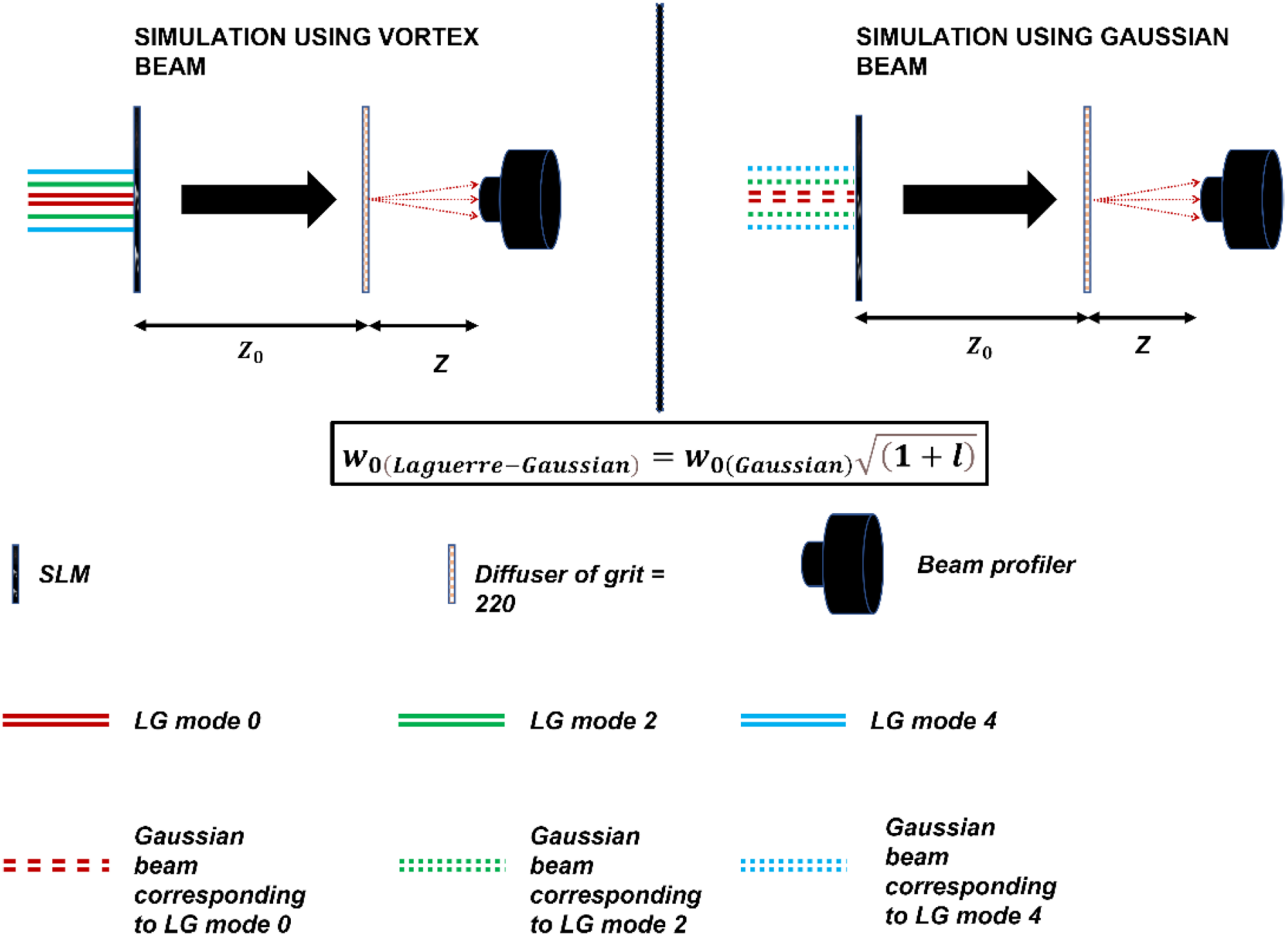
Concept of the imaging setup.

Moreover, recent studies indicate that optical imaging is improved through deep learning algorithms^27–33^. This article proposes a proof-of-concept simulation model and provides experimental verification to enhance imaging through diffuse media using multiple modes of vortex beams and convolution neural networks. The use of vortex beams, coupled with a deep learning algorithm, allows us to gain significant insight into the potential impact of vortex beams to enhance the image reconstruction quality and their potential applications in imaging through diffuse media. These applications include biomedical imaging, imaging through a fog, non-destructive testing, computer-assisted surgery, and autonomous vehicular systems^23,26,34^. In this article, we investigate imaging through diffuse media by means of numerical simulations and experiments. The setup includes a light source (vortex beams), the test object, a diffuser, and a beam profiler or beam profiler. The light from the source is propagated and reflected from the object displayed at the SLM. The reflected light is propagated through the diffuser and received by the beam profiler, as shown in Fig. 1.

The structure of the paper is as follows: “Orbital angular momentum of light and imaging using vortex beams” section describes the theory of vortex beams. “Imaging through scattering media: multi-mode scanning mechanism using vortex beams section describes the optical imaging mechanism. Section explains the “Simulations”. “LGDiffNet: Deep learning architecture for image reconstruction section describes the “LGDiffNet” convoluted neural network (CNN) used in conjunction with the imaging system. The performance analysis of the “LGDiffNet” is shown in “Image reconstruction using “LGDiffNet” CNN architecture” section. The experimental verification and results are shown in “Experimental verification, results, and discussions” section, and we finish with the conclusions in “Conclusions” section.

## Theory and setup design

The theory about vortex beams and the multi-mode scanning mechanism using vortex beams are described in this section.

### Orbital angular momentum of light and imaging using vortex beams.

Conventional laser beams usually have a spherical wavefront where the azimuthal phase or topological charge (*l*) is *l* = 0. However, it is possible to change the wavefront of laser beams by imparting a topological charge. Any beam carrying a topological charge is said to possess orbital angular momentum (OAM). Beams containing OAM have a helical wavefront and are called vortex beams. The Laguerre-Gaussian (LG) are the most common examples of light beams carrying OAM. They are mathematically obtained by solving the paraxial wave equation in the circular cylindrical coordinates^35–38^. Compared to the traditional Gaussian beam, vortex beams in an imaging system show significant improvement in image quality. This is due to the object’s selective illumination by non-overlapping modes of the vortex beams used, which enhances specific parts of the sample, combined digitally to give a complete sample image. Different modes of OAM beams do not interfere with each other in free space propagation. However, it has to be noted that when OAM beams enter diffuse media, the scattering photons move into photons' propagation paths from different modes. Increasing the width of the media or even the scattering phase and amplitude function of the scatterer reduces the number of ballistic and snake photons that reach the beam profiler, which is essential for a beam to hold its shape. At some point, the output pattern no longer resembles the original mode^39^.

The mathematical expression which describes the complex amplitude of the LG beams is given by:

1 $${U}_{mn}^{LG}\left(r,\varphi ,z\right)={C}_{mn}^{LG}\left(\frac{1}{w}\right)\exp\left(\frac{-ik{r}^{2}}{2R}\right)\exp\left(\frac{{r}^{2}}{{w}^{2}}\right)\exp\left(-i\left(n+m+1\right)\Psi \right) \times \exp\left(-i\left(n-m\right)\right)\varphi {L}_{{\min}(n,m)}^{\left|n-m\right|}\left(\frac{2{r}^{2}}{{w}^{2}}\right)$$

Here k is the wavenumber, R is the radius of curvature of the phase front, w is the beam waist of the Gaussian term, m, and n are quantum numbers (Such that n = m + *l* and n + m = 2p + *l* = N. *l*, N, and p are the topological charge, degree, and order respectively). The LG modes are the most accessible type of vortex beams to generate in a laboratory setting. Excellent reviews exist on the theoretical explanation of OAM, generation of LG beams, and applications using LG beams^40,41^. An extensive study of the vortex beams has found ample applications in the field of communications and optical imaging, among others^34,38,42–49^.

### Imaging through scattering media: multi-mode scanning mechanism using vortex beams

This section presents the Gaussian beam and Laguerre-Gaussian beam, which will later be used in our simulation and analysis. The diffuser is designed such that the impulse response of the diffuser to the input light field is given as^16^:

2 $${I}_{{Out}_{\left(i,j\right)}} \alpha F({I}_{{in}_{\left(i,j\right)}}, \lambda ,z,{A}_{obj},{P}_{scat})$$

where ${I}_{{Out}_{(i,j)}}$ is the intensity recorded on the beam profiler, F is the impulse response of the diffuser to the field containing the information about the object, which is a function of the input power, the wavelength of light used, and the phase response of the diffuser and object.${I}_{{in}_{(i,j)}}$ is the input field which is the Gaussian or the Laguerre-Gaussian modes, ${A}_{obj}$ is the phase response of the object behind the diffuser to the input light field, varied between 0 to 2π, and ${{P}_{scat}}_{(i,j)}$ is the phase response of the diffuser which interacts with the field. Here, *i* and *j* are the pixel number values in the x and y-direction, respectively.$\lambda (=632.8\, {\mathrm{nm}})$ is the wavelength of light used and $z ( =2*{10}^{-2}\, {\mathrm{m}})$ is the propagation distance.

**Figure 2:**
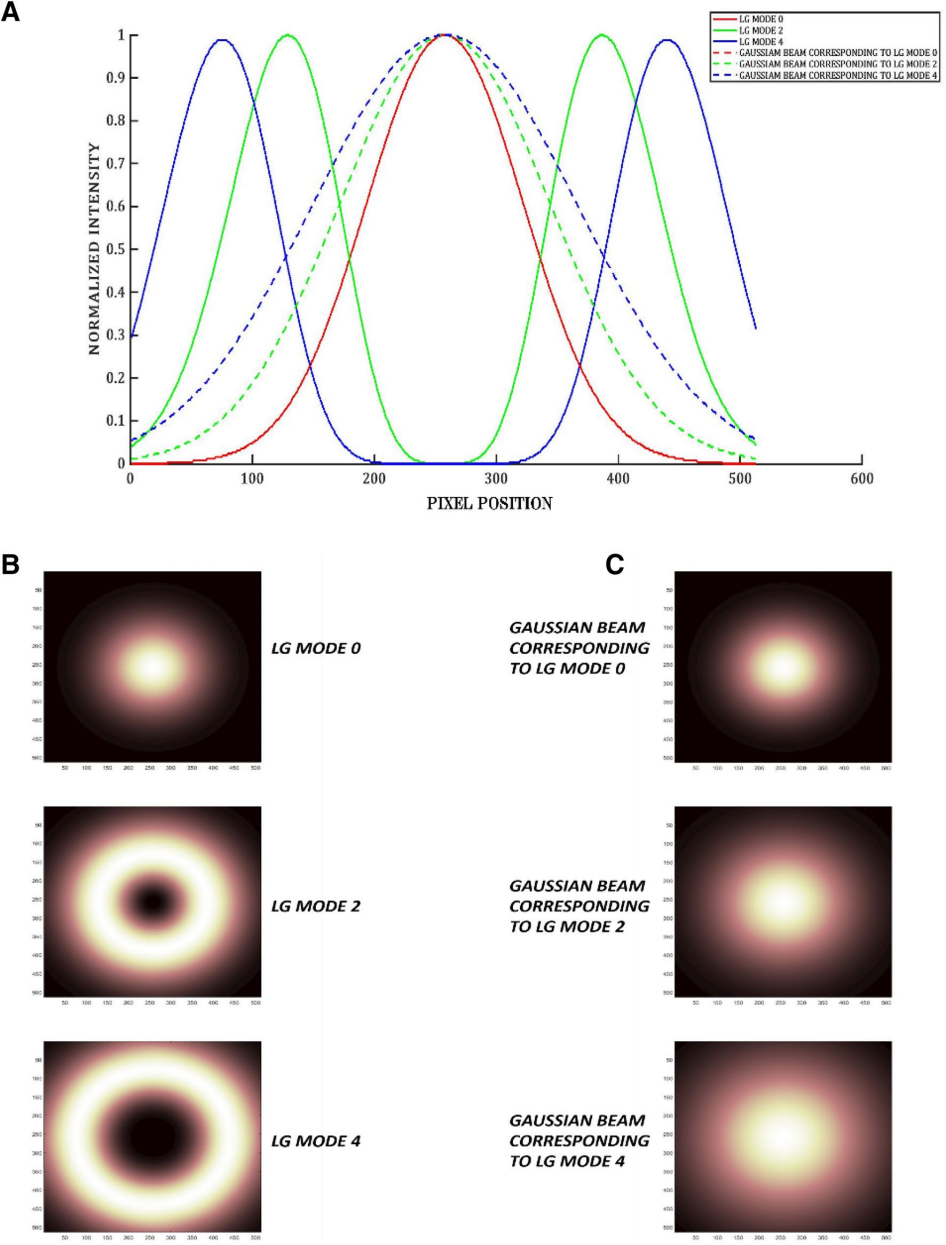
(**A**) Shows the intensity of each simulated LG mode (solid lines) and their corresponding Gaussian beam modes (dotted lines) with respect to the pixel values. (**B**) and (**C**) Show the intensity profile of the LG modes and the corresponding Gaussian beam modes used in the simulations.

For each different mode of the beam, the beam profiler captures a 512 × 512 image. Three modes of vortex beams are used in this article. When the three different beams scan each object, the images captured on the beam profiler were assigned to a 512 × 512 × 3 3-dimensional matrix (see Fig. 2). Therefore, virtually every channel contains information captured for a scan order. In other words, channel 1 contains a scan of the smallest Gaussian beam; channel 2 contains the scan of a second-order LG beam (or a twin Gaussian beam). Channel 3 contains the scan of a fourth-order LG beam (or a twin Gaussian beam). Using this technique, we build a multi-mode image, which visually sees how the information is captured on the beam profiler for each mode. As we can see in Fig. 2A,C), increasing the size of the Gaussian beam reduces the power per unit area of the energy center, so when we increase the mode of the Gaussian beams, we distribute the energy to larger areas of the SLM and the diffuser, significantly reducing the amount of light that is transmitted. Therefore, we get a more blurred image. The main idea here is to find a new way to distribute the energy such that we can illuminate the whole object without the need to use a more powerful laser or extra optical components. So that even after increasing the mode of the beam (size of the beam), enough ballistic and snake photons can be collected at the beam profiler, significantly improving the quality of the image.

## Simulation and experiments

The simulation and the experimental setup used in this study and the proposed imaging methodology are as follows: The input beams have a spot size of 1.26 mm and an input power of 5 mW. $\lambda =632.8\, {\mathrm{nm}}$ is the wavelength of light used and $z =2*{10}^{-2}\, {\mathrm{m}}$ is the propagation distance. The Gaussian (three different beams of varying spot sizes corresponding to the LG mode) and L-G beams are generated using a MATLAB code and first SLM. The generated beams are then passed through a phase screen containing the displayed object generated by the second SLM. The output light from the phase screen, which includes information on the displayed object, which is a number from the digits number dataset^46^, is passed through a simulated diffuser of grit 220, which causes the beams to scatter and create a distorted image on the beam profiler.

### Simulations

The input beams are propagated using the split-step Fourier method^50^. This operation, which is easily executable in MATLAB, is used to numerically solve complicated partial differential equations for which it is difficult to ascertain a general solution. The imaging is performed separately for each different mode of the vortex beams. The three beams are then combined to create the complete pattern containing all the number’s information. The same is repeated using three Gaussian beams with corresponding spot sizes to LG beam modes 0, 2, and 4. Equation 3 gives the spot size of the Gaussian beam corresponding to the vortex beam carrying topological charge (*l*)^51^:

3 $${w}_{0\left(Laguerre-Gaussian\right)}={w}_{0(Gaussian)}\sqrt{\left(1+l\right)}$$

where ${w}_{0}$ is the waist size of the beams used.

After considering different combinations of modes used to image the test object, we use LG beams consisting of three topological charges l = (0, 2, 4) to avoid overlapping modes and reduce the aberrations arising due to it. The imaging system also uses three Gaussian beams with spot sizes calculated according to Eq. (3) to compare the imaging system's performance with the two types of beams.

### LGDiffNet: Deep learning architecture for image reconstruction

We use a convolution-based neural network to classify and reconstruct the displayed numbers from the pattern obtained in the beam profiler. The LGDiffNet learns from images to identify the image that has been inserted before the diffuser. The CNN architecture is based on the encoder-decoder idea of UNet architecture^52^. Although initially, this architecture was used for a segmentation problem, recent studies have shown that this architecture is very efficient for the required restoration^16,53,54^. However, several changes have been made to the original architecture. Firstly, the image input enters three convolution layers, with eight kernels of the size 5 × 5. One layer is dilated equally to 2, and another layer is dilated equally to 3. The three feature maps obtained are connected to get one feature map. The resulting feature maps enter the down sample layer (average-pooling 2 × 2). From this position, the process repeats itself five times in the encoder. The feature map information enters a transition layer consists of batch normalization (BN) layer followed by the ReLU activation layer, then convolution with ${2}^{4+i}$ kernels of size 5 × 5, where $i$ indicates the step number. The feature maps then enter the dense block consisting of 4 convolution layers with 16 kernels of size 5 × 5. Between each of the two convolution layers, there is a BN layer and a ReLU activation layer. In addition, at the entrance to each convolution layer in the block, connections are made with the feature maps at the exit from the previous convolution layers in the block and the feature maps at the entrance to the block via skip connections. At the end of each stage, the feature maps enter into a sample layer (max-pooling 2 × 2). The bottleneck consists of one transition layer and one dense block. Now, the decoder part starts with five steps corresponding to the five that were in the encoder. The output from the bottleneck then enters the up-sample layer (Transpose convolution 2 × 2). To preserve the information, skip connections are performed with the feature maps obtained at the end of the corresponding phase in the encoder with the feature maps obtained after the up-sample layer. This feature map is then mapped to the transition layer and from there to the dense block (dense block and transition layer are the same as the decoding phase). The CNN also performs an up-sample once more and completes another convolution operation to obtain the network output. It has been recently shown that using a dilated convolution layer and convolution layer with a wide kernel allows better information extraction from images^16^. Additionally, the dense-net method has been shown to improve network performance for better convergence^55^.

Since the network solves a regression problem, adjusting the regression problem’s loss is essential for optimal network performance. Although Mean Square Error (MSE) and Mean average error (MAE) loss functions are typical for the regression problem, studies have shown that they are less suitable for the problem as the input images do not offer a perfect resemblance to the ground truth. We, therefore, chose to use the Negative Pearson Correlation Coefficient (NPCC) loss function, which is effective for this type of problem^16,32,56–58^:

4 $$NPCC=\frac{\sum_{i=1}^{W}\sum_{j=1}^{H}\left(Y\left(i,j\right)-\tilde{Y }\right)(G\left(i,j\right)-\tilde{G })}{\sqrt{\sum_{i=1}^{W}\sum_{j=1}^{H}{\left(Y\left(i,j\right)-\tilde{Y }\right)}^{2}}\sqrt{\sum_{i=1}^{W}\sum_{j=1}^{H}{\left(G\left(i,j\right)-\tilde{G }\right)}^{2}}}$$

W and H are the respective comparator image’s width and height, G is the ground truth, and Y is the CNN output. $\tilde{G}$ and $\tilde{Y}$ are the mean values of ground truth and CNN output, respectively. The CNN was built and trained on a computer with an i9 series 9900 k processor, two NVIDIA GeForce RTX 2080Ti graphics processors. Each GPU has a VRAM of 11 GB, and an NVlink is provided between the two GPUs. The network was trained for 40 epochs by Adam Optimizer with an initial learning rate of 0.0001, which reduces by half after ten epochs. The network was trained two times, once for the beam-profiler recorded images for the Gaussian beams and once for the beam profiler-recorded pictures for the different modes of LG beams.

**Figure 3:**
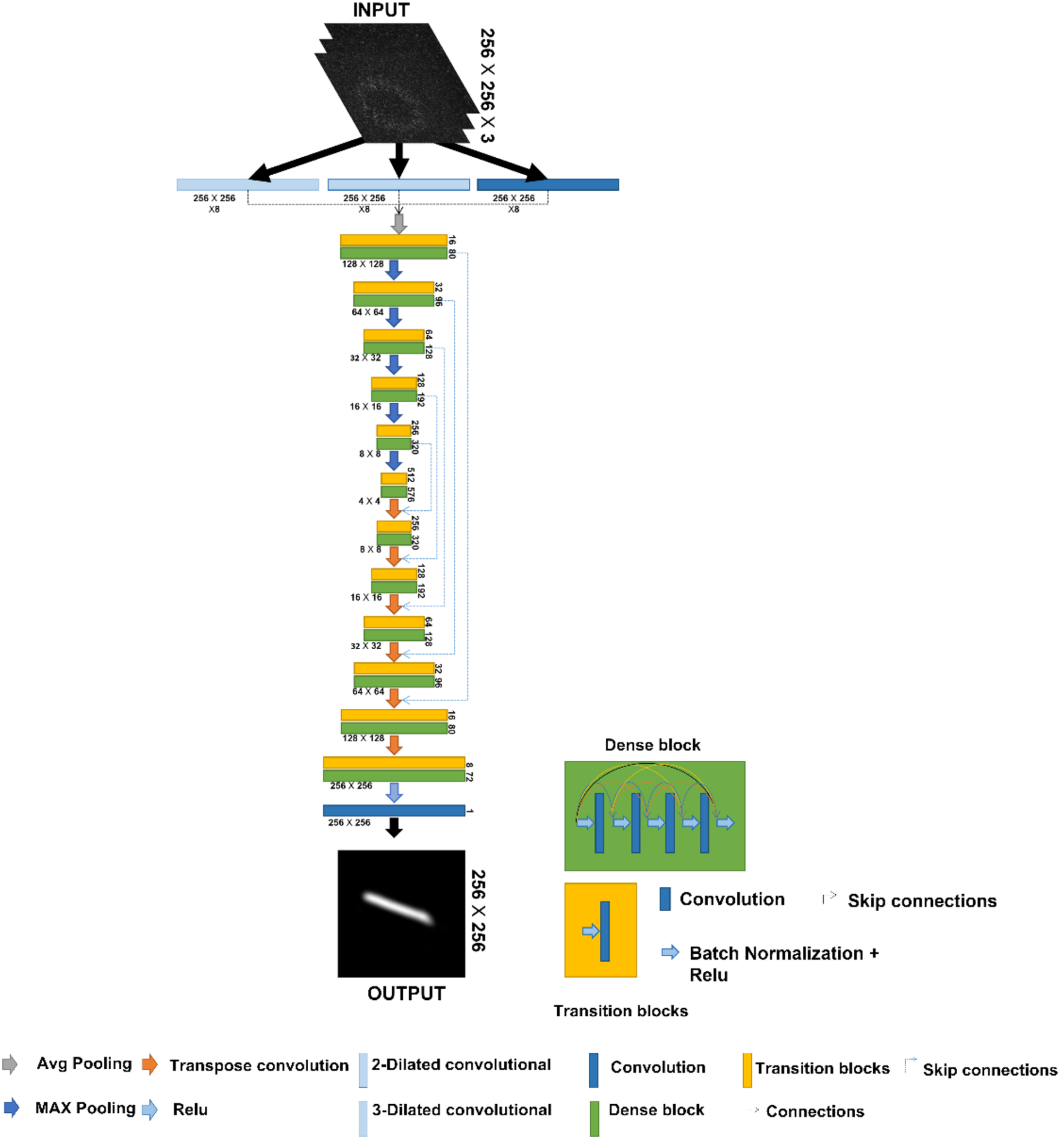
Block diagram of the LGDiffNet network architecture.

Supervised convoluted learning requires a lot of labeled data to train the learning parameters well; thus, we used a data set containing an extensive collection of different images. To train and examine the network, we used a Digits data set containing 10,000 images of handwritten numbers from 0 to 9 (1000 for each number), where a certain angle rotates each image^59^. Each image is of the size 28 × 28 pixels, containing pixel values from 0 to 255. Each image is resized to a 512 × 512 pixel resolution to match the image resolution to the beam profiler resolution. We rescaled the pixel values according to the phase change value that each pixel contributed to the beam. The original image was used as ground truth labels for training purposes. Our simulation gives us the images recorded in a 512 × 512 resolution beam profiler. In this simulation, the beam profiler’s pixel size and the number’s images are 6.45 µm. Due to computational limitations, the images captured on the beam profiler have been downsampled to 256 × 256 pixels, leading to loss of information as the downsample algorithm averages 2 × 2 neighboring pixels. Figure 3 shows the network structure used in the manuscript.

### Image reconstruction using “LGDiffNet” CNN architecture

**Figure 4:**
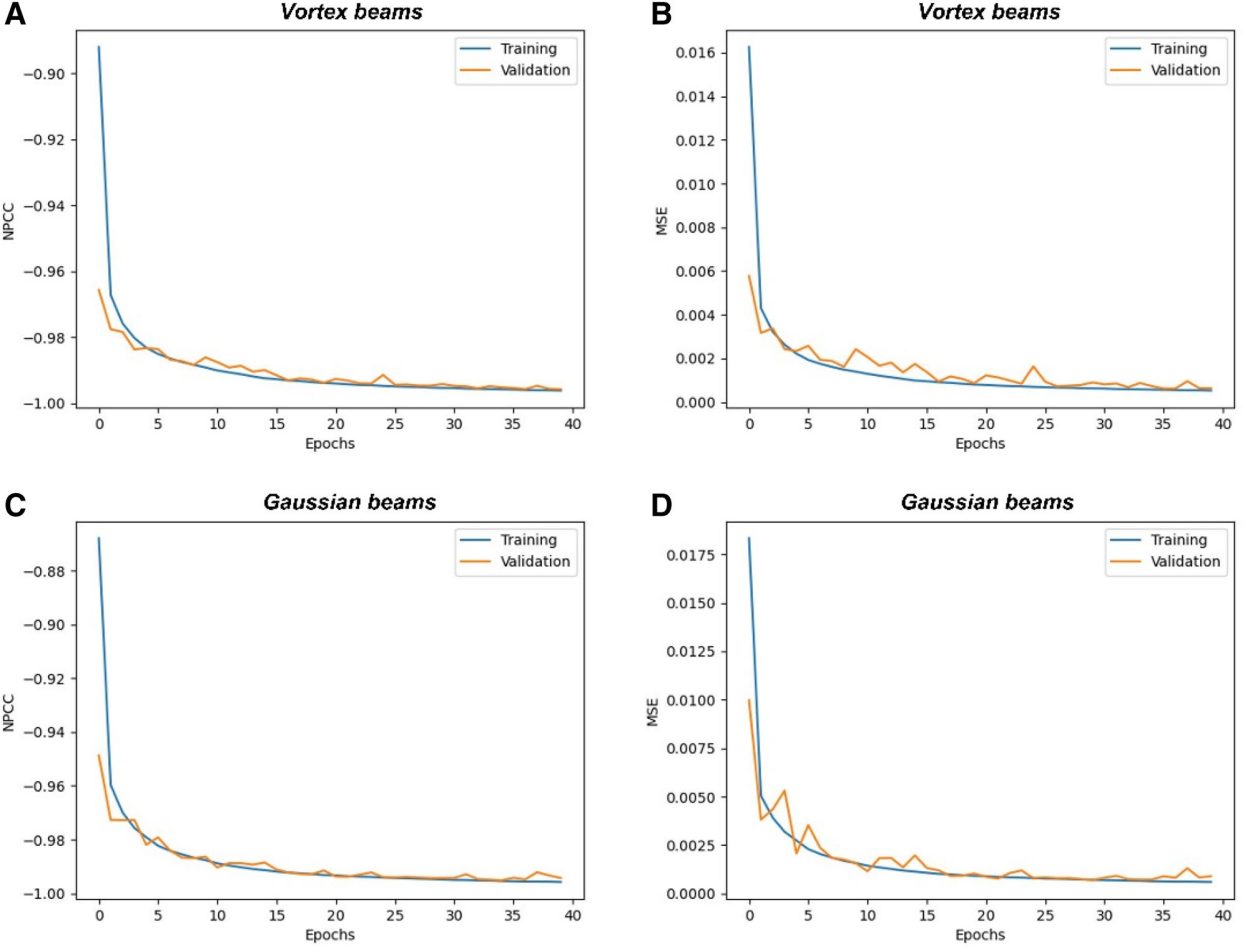
Shows the training and validation convergence graphs for the CNN used in the study. The convergence of the NPCC function for the image reconstruction process for both the vortex beams (**A**,**B**) and Gaussian beams (**C**,**D**) is shown. The reduction in the mean squared error (MSE) with each epoch is also shown.

**Figure 5:**
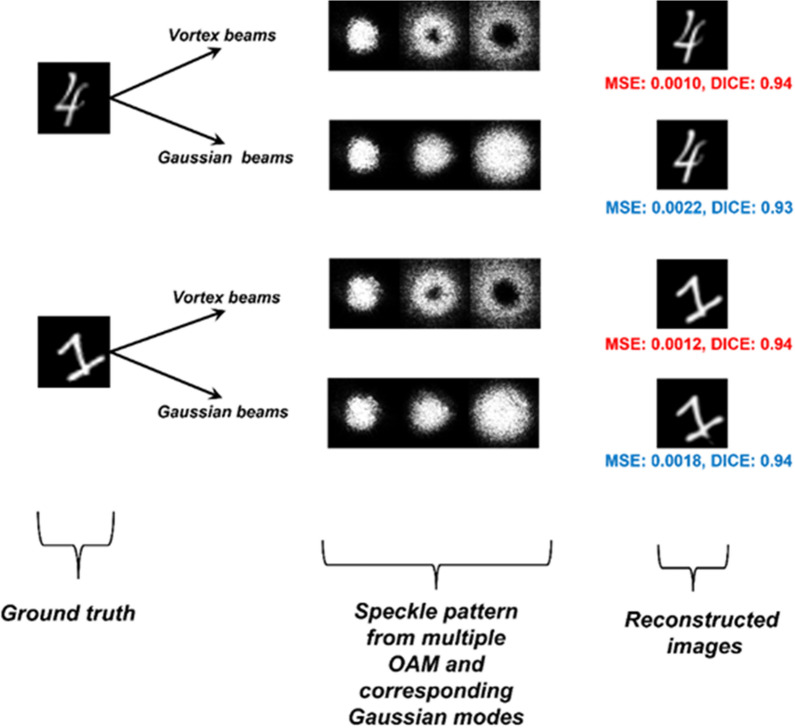
The patterns from the vortex beams and the Gaussian beams and the reconstructed images using the CNN are shown.

In this section, we present the training and validation of the CNN algorithm detailed in the "LGDiffNet: Deep learning architecture for image reconstruction" section. As described in “Orbital angular momentum of light and imaging using vortex beams” section, a diffuser of grit = 220 is used to simulate the beam propagation in a diffuse media, and the images are obtained for the three modes of vortex beams. The CNN is trained for 40 epochs in each iteration. The NPCC loss function is used in the algorithm. With each passing epoch, the algorithm learns more from the image training set, which is comprised of 90% of all the images obtained. From the training set, 10% of the images are randomly selected for validation. 10% of all the images are used for testing the algorithm. The training results and tests are shown in Figs. 4 and 5.

Figure 4 shows the training graphs for the convergence of the NPCC function with each epoch for a diffuser of grit = 220. Comparatively, the LG beams perform much better in terms of image resolution of reconstructed images. This high-performance by the CNN when vortex beams are used can be attributed to the orthogonality of the different LG modes and also the distribution of the intensity patterns in the vortex beams with respect to the Gaussian beams. The mean squared errors for the image reconstruction are 0.0008 when Gaussian beams are used as compared to 0.0006 when vortex beams are employed. The training and validation processes are also considerably smoother and more robust when vortex beams are used, which can be observed from the training and validation graphs shown in Fig. 4.

The patterns obtained from the simulations and the computational image reconstruction using the LGDiffNet convolution neural network are shown in Fig. 5. The NPCC for image reconstruction using vortex beams is − 0.9956 compared to the NPCC of − 0.9942 when Gaussian beams are used for imaging acquisition. The Sørensen-Dice score is also applied to the reconstructed images to check for the validity of the image reconstruction algorithm and the imaging system itself^60^. The vortex beams still show an enhancement in the reconstructed images in this regard. The calculated peak signal-to-noise ratio (PSNR) increases by ~ 1 dB when vortex beams are used. The images reconstructed when the image is obtained using vortex beams have a PSNR of 80.34 dB compared to 79.09 dB when the images are reconstructed using images from the Gaussian beams.

The results shown here are, however, theoretically simulated. To check for the actual enhancement caused by vortex beams compared to Gaussian beams, we conduct an experiment to verify the results obtained by simulations. The experimental procedure and the corresponding results are described in the next section.

### Experimental verification, results, and discussions

**Figure 6:**
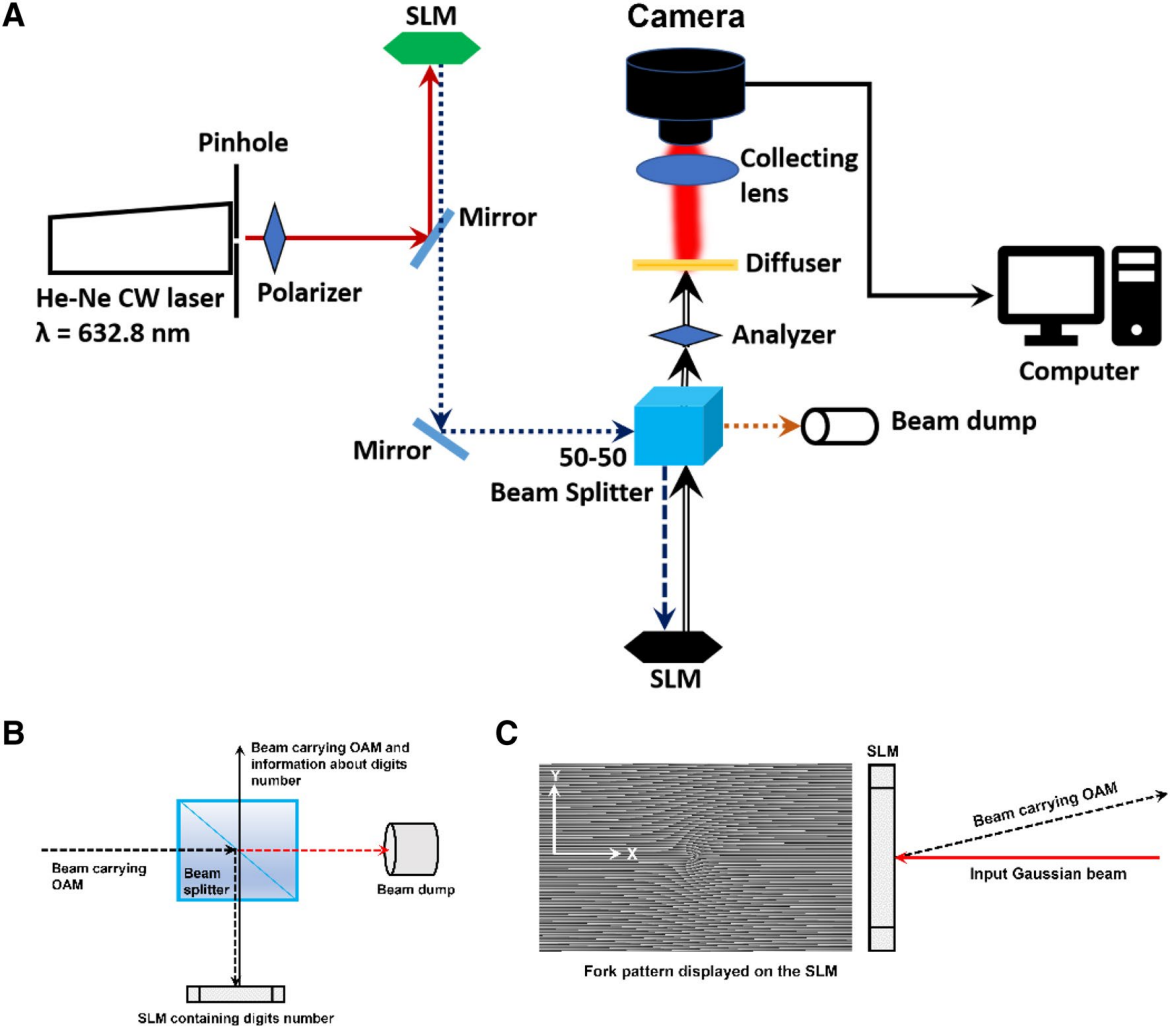
(**A**) Experimental setup used to verify the enhancement caused by illuminating the diffuser with different modes of vortex beams. (**B**) Propagation of the vortex beams through the beam splitter. (**C**) Imparting the topological charge to the Gaussian beams along with the tilt using a forked hologram to cause a shift in the propagation axis.

The experimental imaging setup is shown in Fig. 6A. A laser beam of λ = 632.8 nm provides illumination. The beam is emitted from a Melles-Griot 05-LHP-123–496 He–Ne laser system. The input beam power is measured to be 5 mW, and the laser beam has a spot size of 1 mm. The beam is then passed through a polarizer and directed towards the SLM's (HOLOEYE PLUTO 2.1) center using an aluminum mirror. The SLM, which has a resolution of 1920 × 1080, pixel size of 8 µm, and a frame rate of 60 Hz, displays a fork pattern that imparts orbital angular momentum to the input laser beam and also shifts the beam upwards in the y-direction (see Fig. 6B). The laser beam carrying OAM is then passed through a 50:50 beam splitter (BS) with the help of another mirror. Half of the emitted beam from the BS is collected at the beam dump, and the other half is incident on another SLM (Jasper LCoS) that contains the displayed digits dataset number (see Fig. 6C). The Jasper SLM has a resolution of 1920 × 1080, a pixel size of 6.45 µm, and a frame rate of 60 Hz.

The reflected beam from the second SLM is then passed through a diffuser of grit 220 (Thorlabs DG05-220) via an analyzer. Diffusers are optical windows that scatter light and produce a diffusive speckle pattern. The scattering of light passing through diffusers is caused due to the correlation between the incident field and the diffuser surface's hills and valleys^61^.The Optical depth and the optical mean free path of the diffuser are calculated using the reference^62^, and described in the supplemental document.From the experimental calculations, we determine that the diffuser of width 2 mm is 7.11 times the optical mean free path of the sample.

**Figure 7:**
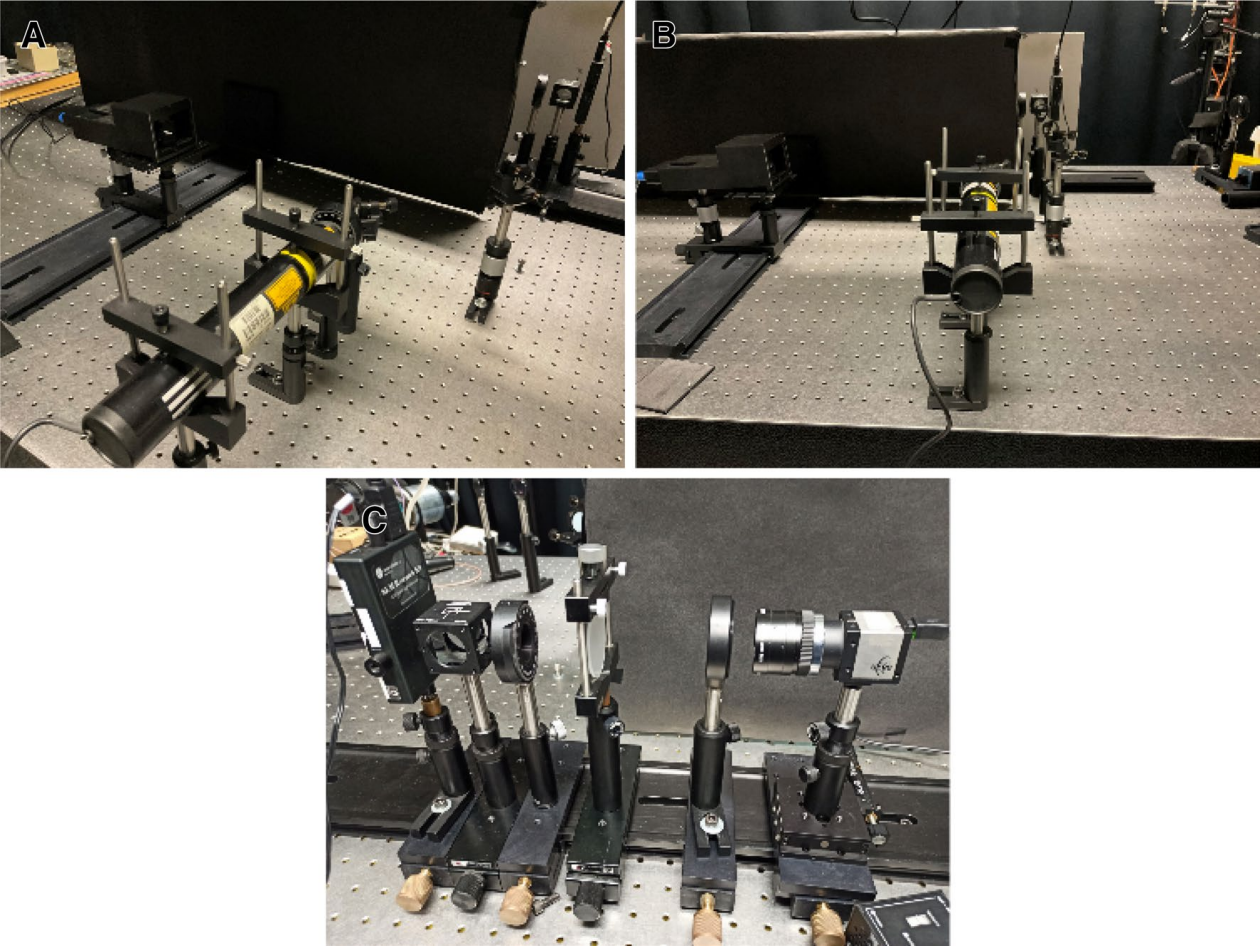
(**A**) and (**B**) Show the imaging setup of the imaging with multiple vortex modes and their corresponding Gaussian modes, respectively. (**C**) Shows the SLM displaying the digits dataset numbers^63^ and the image acquisition setup.

A beam profiler (uEYE UI-2210-C, 640 × 480 pixels with a pixel size of 10 µm) then collects the resultant pattern caused due to the diffuser, with the help of a telescopic lens. Since the diffuse media emits light in all directions, the beam profiler was placed as close as possible (~ 2 cm) to the diffuser. The images are acquired for modes *l* = 0(*channel* 1), 2(*channel* 2), 4(*channel* 3), and they are digitally combined to provide a complete pattern. The modes are also given a color-coding in the computer to identify them in the images. The same is repeated for three corresponding Gaussian beams (as shown in “Orbital angular momentum of light and imaging using vortex beams” section). However, in this case, a 4-*f* lens setup is introduced before the beam splitter to attain magnifications to the beam, which are approximately corresponding to the spot sizes of LG modes (see Eq. 3.). The photo of the experimental setup is shown in Fig. 7.

**Figure 8:**
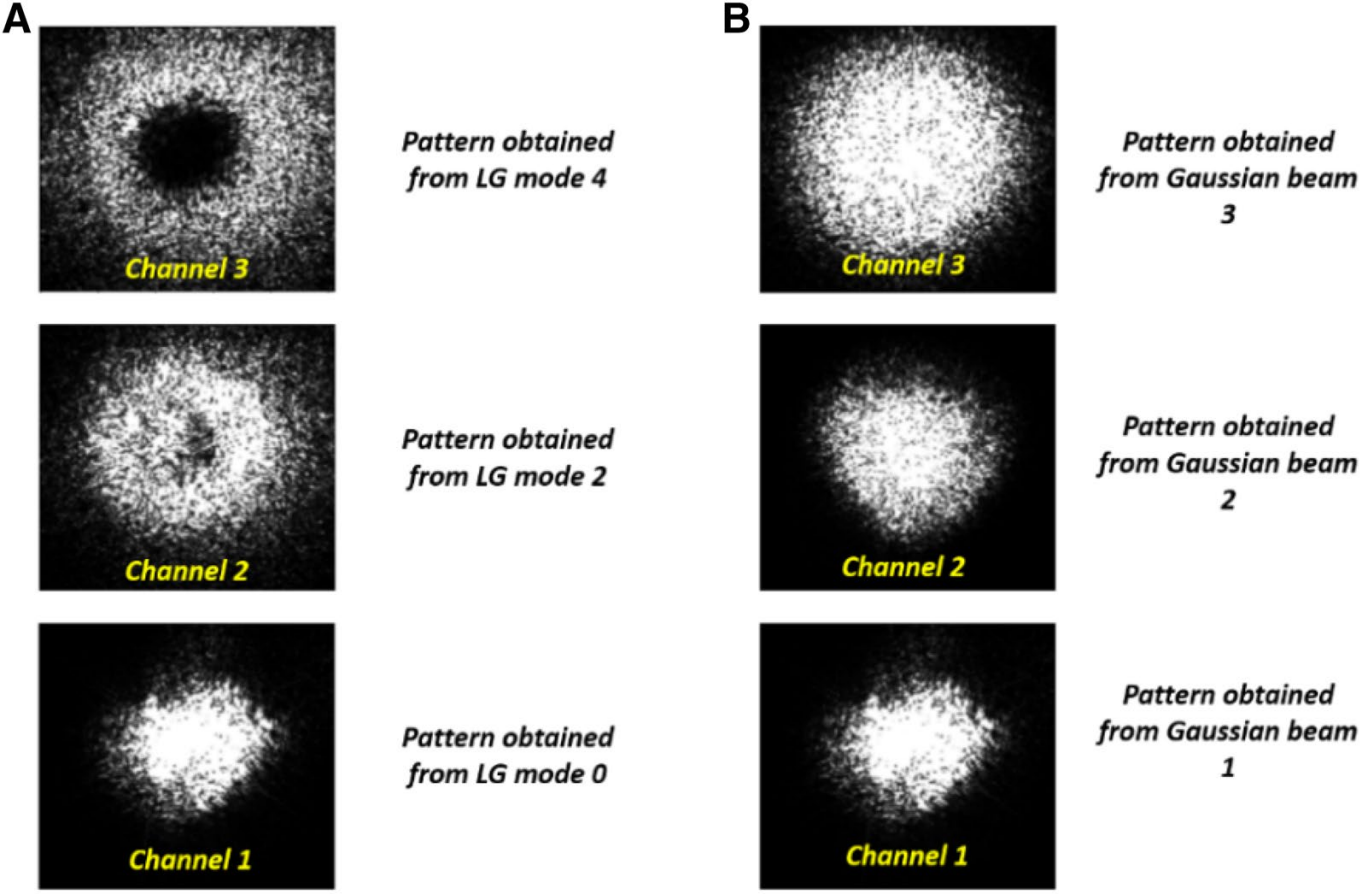
(**A**) and (**B**) Show the multimodal images captured by the beam profiler of the LG modes and the corresponding Gaussian beam modes in the experiment. The changes in the contrast of the images as compared to the simulated images is due to the difference in the colorbar and exposure settings of the beam profiler.

**Figure 9:**
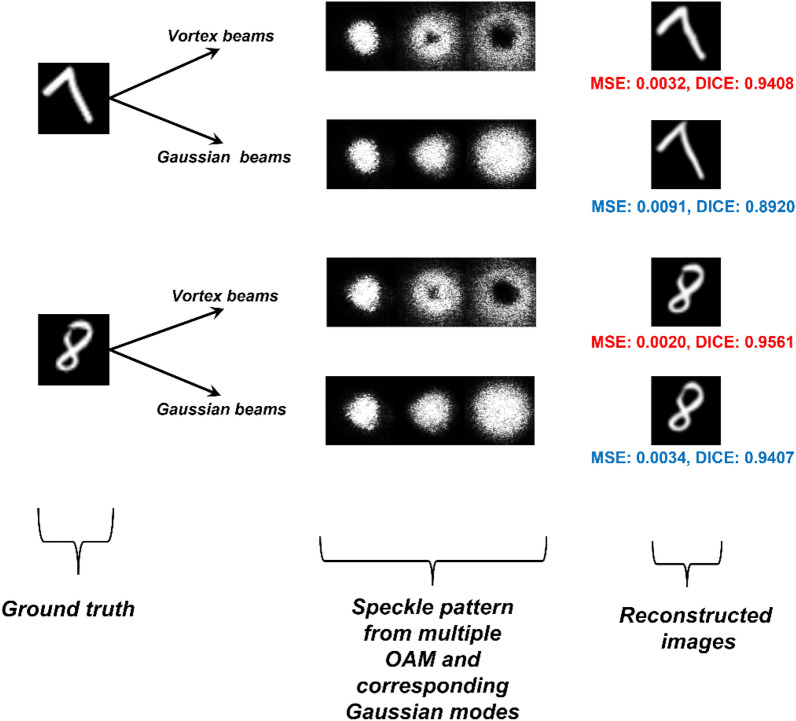
The experimental patterns from the vortex beams and the Gaussian beams and the reconstructed images using the CNN are shown. The changes in the contrast of the images as compared to the simulated images is due to the difference in the colorbar and exposure settings of the beam profiler.

**Figure 10:**
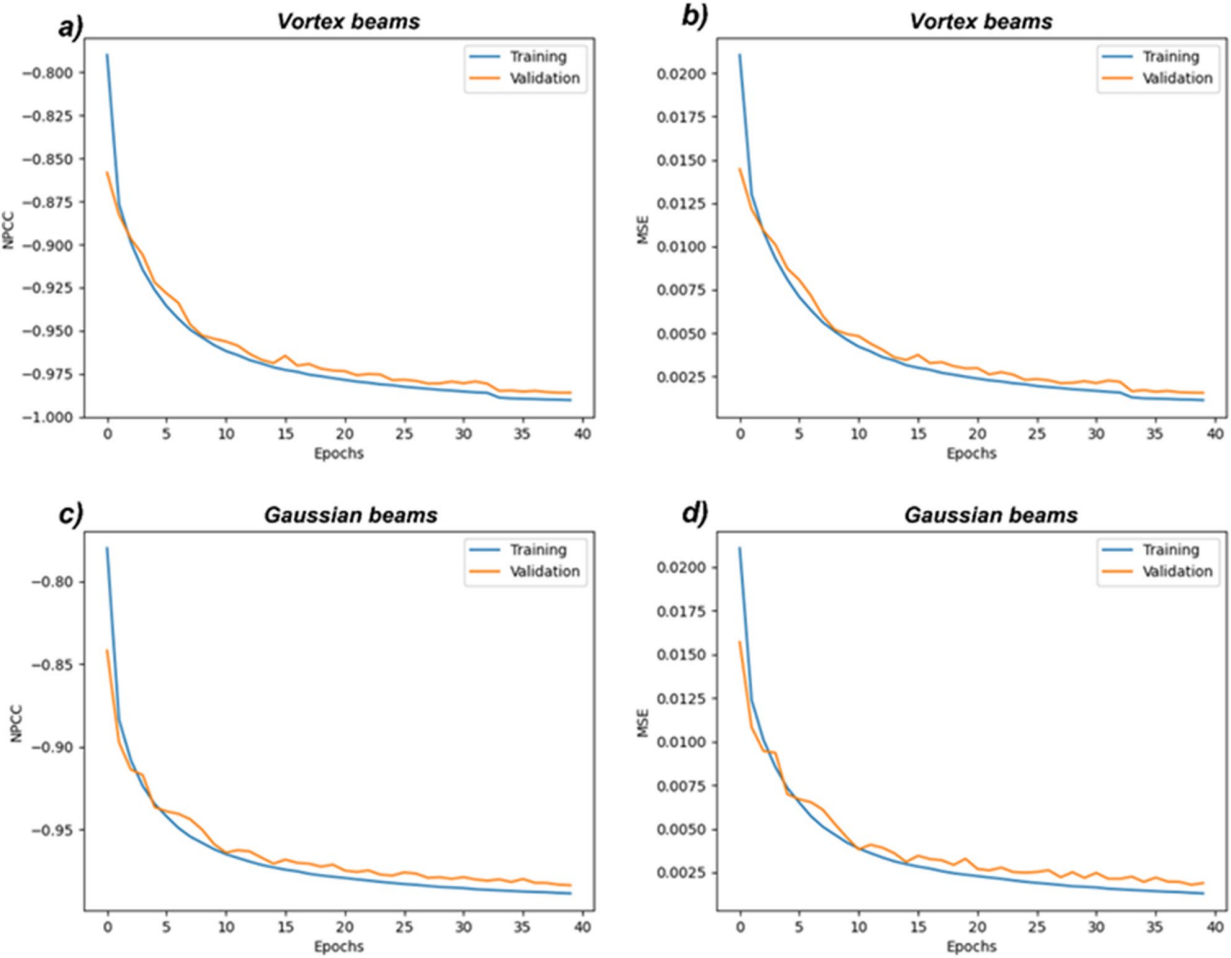
Shows the training and validation convergence graphs for the CNN used in the study. The convergence of the NPCC function for the image reconstruction process for both the vortex beams (**A**,**B**) and Gaussian beams (**C**,**D**) is shown. The reduction in the mean squared error (MSE) with each epoch is also shown.

The acquired images (see Fig. 8) are sent to the LGDiffNet CNN algorithm, and the results are shown in Fig. 9. Like the simulation results, the imaging system using multiple vortex beam modes outperforms the imaging system using various Gaussian beams corresponding to the LG mode. The best NPCC for image reconstruction using vortex beams is − 0.9850 compared to the best NPCC of − 0.9837 when Gaussian beams are used for imaging acquisition. The vortex beams also show an enhancement in terms of the Dice coefficient, where the images reconstructed using data acquired from vortex beams have a score of 94.7% compared to 94.27% when data from the Gaussian beam are used. The calculated peak signal-to-noise ratio (PSNR) also increases by 0.62 dB when vortex beams are used. The images reconstructed when the image is obtained using vortex beams have a PSNR of 76.50 dB compared to 75.88 dB when the images are reconstructed using images from the Gaussian beams. Figure 10 shows the training graphs for the convergence of the NPCC function with each epoch when a diffuser of grit = 220 is used.

Having shown the experimental results, we find that they agree well with the theory and numerical simulations. Several parameters like the NPCC and MSE are used to test the validity of the experiment and simulations. The PSNR is subsequently calculated from the MSE. Firstly, in terms of the parameters shown in Table 1, we see that there is < 1% deviation in calculated values for the theory and experiments. They show that the experimental and simulated results are in accordance with each other. The minor deviations may be caused due to many factors, including imperfections in the optics and the sample, the loss of beam power and shape due to its interactions with the optics. Secondly, the enhancement shown by the calculations suggests that there is always an increment in the PSNR and other calculated parameters when three channels of vortex beams are used in imaging instead of three channels of Gaussian beams. Finally, the high values of PSNR demonstrate the incredible robustness and adaptability of the LGDiffNet CNN architecture, which is significant because no additional optimization methods or reference beams are used. However, it should be noted that the performance of the proposed neural network depends heavily on the initialization of the hyperparameters used in the training phase. Since the training batches are always selected at random from the training data, variations are to be expected in the validation results, depending on the weights and biases acquired from the training process and the training dataset. Moreover, different types of neural networks and datasets using the same optical method could greatly vary the results depending on the complexity of the neural network and the datasets.

**Table 1:** Image enhancement using vortex beams and deep learning.

Parameters	Imaging using Gaussian beams	Imaging using vortex beams
MSE	Simulation: 0.0008	Simulation: 0.0006
	Experiment: 0.0018	Experiment: 0.0016
PSNR (dB)	Simulation: 79.09 dB	Simulation: 80.34 dB
	Experiment: 75.88	Experiment: 76.50
NPCC	Simulation: − 0.9942	Simulation: − 0.9959
	Experiment: 0.9837	Experiment: 0.9850
Sørensen-Dice coefficient	Simulation: 0.961	Simulation: 0.970
	Experiment: 0.9429	Experiment: 0.9477

## Conclusions

In this article, we investigated whether illuminating diffuse media using multiple modes of vortex beams instead of Gaussian beams improves the imaging capability of the imaging system and enhances the reconstruction ability of the CNN. A novel CNN architecture called “LGDiffNet” is also developed in this study for image reconstruction. Both numerical and experimental analyses are performed. The results, which have a < 1% deviation between simulation and experiment, show that illuminating diffusers using multiple modes of vortex beams enhances the imaging system and improves the reconstruction ability of the CNN architecture. The various parameters used to check for the improvement, and the enhancement shown in these parameters when vortex beams are used, are shown in Table 1.

In conclusion, a robust and computationally efficient imaging system using multiple modes of vortex beams and the “LGDiffNet” architecture is developed. The results suggest that the imaging system that uses beams carrying topological charges, combined with a CNN to reconstruct the images, has numerous medical imaging and microscopy applications, among others^34,50,64–66^.

## Supplementary Information

Supplementary Information 1.

Supplementary Information 2.

## Data Availability

Data underlying the results presented in this paper are not publicly available at this time but may be obtained from the authors upon reasonable request.
