# Author Correction: Imaging through diffuse media using multi-mode vortex beams and deep learning

**DOI:** 10.1038/s41598-022-11108-9

**Published:** 2022-05-10

**Authors:** Ganesh M. Balasubramaniam, Netanel Biton, Shlomi Arnon

**Affiliations:** Department of Electrical and Computer Engineering, Ben-Gurion University of the Negev, 8441405 Beersheba, Israel

Correction to: *Scientific Reports* 10.1038/s41598-022-05358-w, published online 28 January 2022

The original version of this Article contained an error in the Abstract.

“An enhancement of ~ 1 dB, in terms of PSNR, is achieved using this method when a highly scattering diffuser of grit 220 and width 2 mm (7.11 times the mean free path) is used.”

now reads:

“When employing vortex beams for image reconstruction, the best NPCC is − 0.9850. However, when using Gaussian beams for imaging acquisition, the best NPCC is − 0.9837. An enhancement of 0.62 dB, in terms of PSNR, is achieved using this method when a highly scattering diffuser of grit 220 and width 2 mm (7.11 times the mean free path) is used.”

Additionally, in the Experimental verification, results, and discussions section,

“The acquired images (see Fig. 8) are sent to the LGDiffNet CNN algorithm, and the results are shown in Fig. 9. Like the simulation results, the imaging system using multiple vortex beam modes outperforms the imaging system using various Gaussian beams corresponding to the LG mode. The NPCC for image reconstruction using vortex beams is − 0.9826 compared to the NPCC of − 0.9802 when Gaussian beams are used for imaging acquisition. The vortex beams also show an enhancement in terms of the Dice coefficient, where the images reconstructed using data acquired from vortex beams have a score of 94.3% compared to 93.7% when data from the Gaussian beam are used. The calculated peak signal-to-noise ratio (PSNR) also increases by ~ 1 dB when vortex beams are used. The images reconstructed when the image is obtained using vortex beams have a PSNR of 75.57 dB compared to 74.51 dB when the images are reconstructed using images from the Gaussian beams. Figure 10 shows the training graphs for the convergence of the NPCC function with each epoch when a diffuser of grit = 220 is used.”

now reads:

“The acquired images (see Fig. 8) are sent to the LGDiffNet CNN algorithm, and the results are shown in Fig. 9. Like the simulation results, the imaging system using multiple vortex beam modes outperforms the imaging system using various Gaussian beams corresponding to the LG mode. The best NPCC for image reconstruction using vortex beams is − 0.9850 compared to the best NPCC of − 0.9837 when Gaussian beams are used for imaging acquisition. The vortex beams also show an enhancement in terms of the Dice coefficient, where the images reconstructed using data acquired from vortex beams have a score of 94.7% compared to 94.27% when data from the Gaussian beam are used. The calculated peak signal-to-noise ratio (PSNR) also increases by 0.62 dB when vortex beams are used. The images reconstructed when the image is obtained using vortex beams have a PSNR of 76.50 dB compared to 75.88 dB when the images are reconstructed using images from the Gaussian beams. Figure 10 shows the training graphs for the convergence of the NPCC function with each epoch when a diffuser of grit = 220 is used.”

“Finally, the high values of PSNR demonstrate the incredible robustness and adaptability of the LGDiffNet CNN architecture, which is significant because no additional optimization methods or reference beams are used.”

now reads:

“Finally, the high values of PSNR demonstrate the incredible robustness and adaptability of the LGDiffNet CNN architecture, which is significant because no additional optimization methods or reference beams are used. However, it should be noted that the performance of the proposed neural network depends heavily on the initialization of the hyperparameters used in the training phase. Since the training batches are always selected at random from the training data, variations are to be expected in the validation results, depending on the weights and biases acquired from the training process and the training dataset. Moreover, different types of neural networks and datasets using the same optical method could greatly vary the results depending on the complexity of the neural network and the datasets.”

Furthermore, this Article contained an error in Figure 5 and Figure 10 where data was missing. The original Figure 5, Figure 10 and accompanying legends appear below.

**Figure 5:**
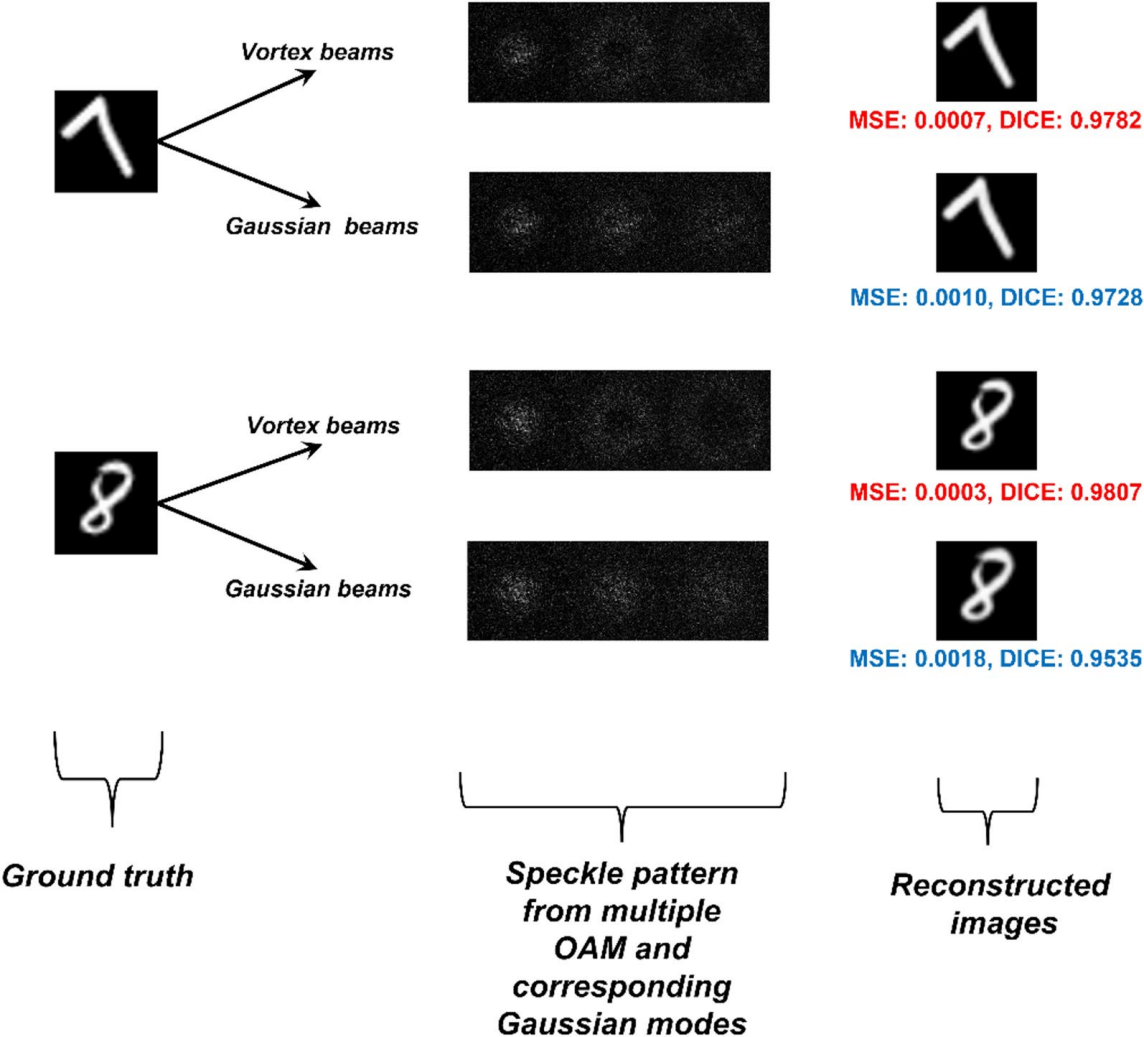
The patterns from the vortex beams and the Gaussian beams and the reconstructed images using the CNN are shown.

**Figure 10:**
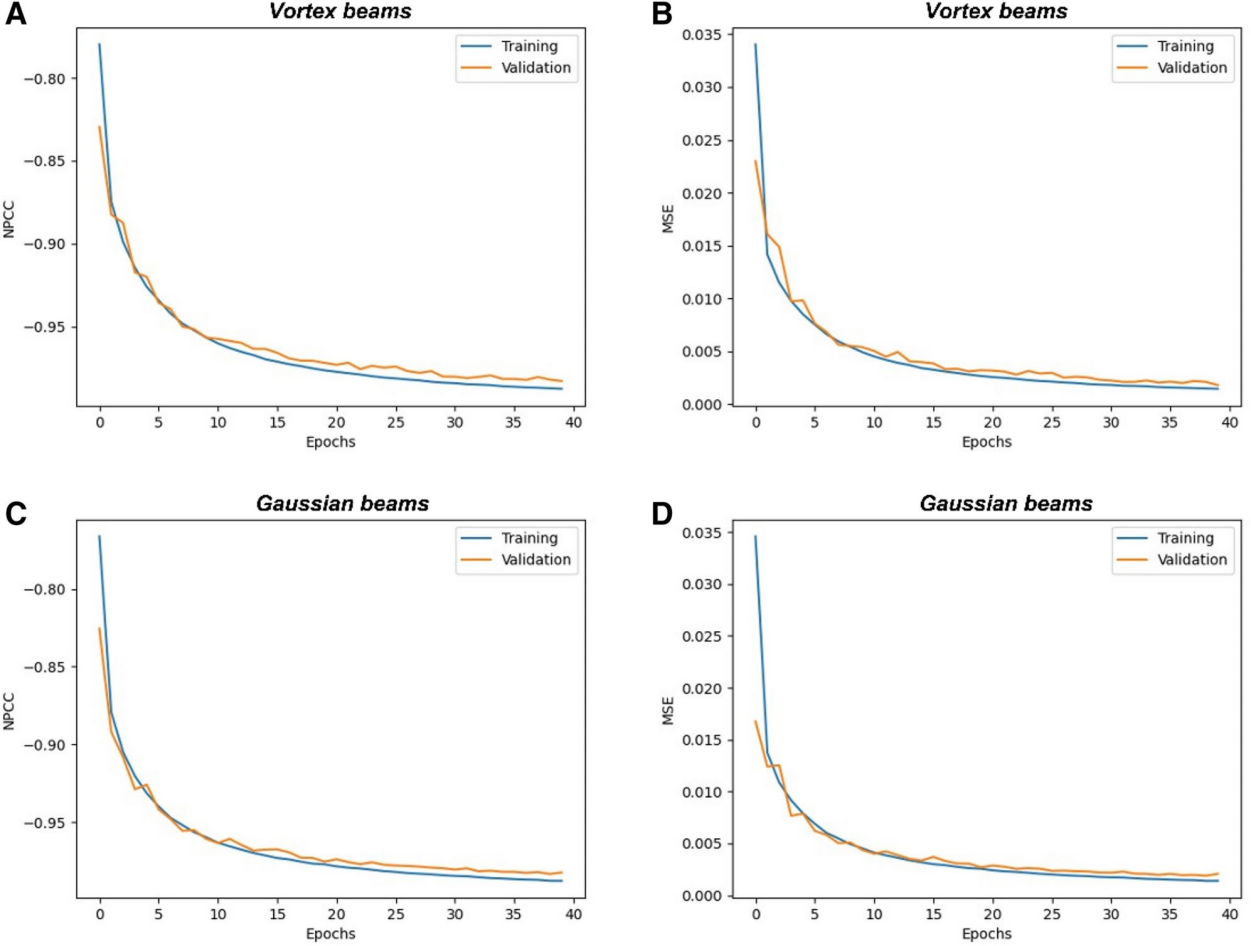
Shows the training and validation convergence graphs for the CNN used in the study. The convergence of the NPCC function for the image reconstruction process for both the vortex beams (**A**,**B**) and Gaussian beams (**C**,**D**) is shown. The reduction in the mean squared error (MSE) with each epoch is also shown.

In Table 1, the Experiment values for “Imaging using Gaussian beams” and “Imaging using vortex beams” were incorrect for the Parameters “MSE”, “PSNR”, “NPCC” and “Sørensen-Dice coefficient”. The original Table 1 and accompanying legend appear below.

**Table 1:** Image enhancement using vortex beams and deep learning.

Parameters	Imaging using Gaussian beams	Imaging using vortex beams
MSE	Simulation: 0.0008	Simulation: 0.0006
	Experiment: 0.0023	Experiment: 0.0018
PSNR (dB)	Simulation: 79.09 dB	Simulation: 80.34 dB
	Experiment: 74.51 dB	Experiment: 75.57 dB
NPCC	Simulation: − 0.9942	Simulation: − 0.9959
	Experiment: − 0.9802	Experiment: − 0.9826
Sørensen-Dice coefficient	Simulation: 0.961	Simulation: 0.970
	Experiment: 0.937	Experiment: 0.943

Finally, Supplementary Information 2 was omitted. The Supplementary Information 2 file now accompanies the original Article.

The original Article has been corrected.

