# Supplementary material for: Imaging through diffuse media using multi-mode vortex beams and deep learning
Source: Sci Rep. 2022 Jan 28;12:1561. doi: 10.1038/s41598-022-05358-w (PMC8799672; doi:10.1038/s41598-022-05358-w)
Supplement: Supplementary file 1 — Supplementary Information 1. [file 41598_2022_5358_MOESM1_ESM.docx]

Imaging through diffuse media using multi-mode vortex beams and deep learning: Supplemental document

This supplemental document establishes a relation between the light scattering due to glass diffusers and the light interaction with tissues. An experiment is also performed to determine the mean free path and the optical depth of the diffuser.

The scattering properties of a diffuser are described in section S.1. We then determine the optical mean free path and the optical depth of the diffuser using an experimental setup described in section S.2.

S.1 Light scattering by a diffuser

Ground glass diffusers, usually 1.5 to 2 mm in width, have a thickness corresponding to several mean free paths (0.03 to 0.4 mm) which is similar to the tissue mean free path [1–3]. Therefore, due to multiple interactions, diffuse photons cause the noise like speckle patterns provided that the sample is thick enough (several mean free paths) to allow multiple scattering events [1,2]. In reference [1], a polystyrene slab of 3 mm thickness (mean free path = 0.22 mm) was used to show deep learning-based lensless imaging through scattering media with potential applications in the biomedical field. Moreover, in reference [4], a close relationship was established between the memory effect region of eggshell membranes and glass diffusers of grit 120 (15 mrad), showing that the correlations between the highly scattered photons in glass diffusers and biological membranes is comparable.

Similar experiments have also been conducted where the scattering and speckle patterns caused by diffusers are found to be similar to a chicken breast slice placed in between two glass slabs ( mean free path = 0.03 mm) [3]. Furthermore, from reference [5], it is clear that a deep learning neural network can be trained using multiple diffusers, and a correlation can be established between the diffusers to deliver optimal performances. Therefore, numerical and experimental analysis can be designed in the medical field by adapting this technique.

The following section determines the mean free path and the optical depth of a ground glass diffuser of width 2mm and grit 220.

S.2 Determining the mean free path of the diffuser: Theory and experiment

The intensity of the light scattered through the diffuser (I) is related to the input intensity of the laser ($I_{0}$) by the equation  [2]:

$$I=I_{0}e^{-\mu_{t}D} (S1)$$

Where D is the width of the diffuser, and $\mu_{t}$ is called the transport coefficient defined by:

$$\mu_{t}=\mu_{a}+\mu_{s}^{'} (S2)$$

$\mu_{a}\mathrm{and}\mu_{s}^{'}$ being the absorption coefficient and the scattering coefficient, respectively.

From equation S1, we get:

$$\mu_{t}=-\frac{ln\left( \frac{I}{I_{0}} \right)}{D} (S3)$$

The optical depth ($d_{s}$) of the diffuser is given by:

$$d_{s}=\mu_{t}D=- ln\left( \frac{I}{I_{0}} \right) (S4)$$

The optical mean free path ($\Lambda$) of the diffuser is given by:

$$\Lambda=\frac{1}{\mu_{t}}=\frac{D}{d_{s}} (S5)$$

The values I and $I_{0}$ are measured to calculate the values of $d_{s}$ and $\Lambda$ using the experimental setup shown in figure S1 [2].


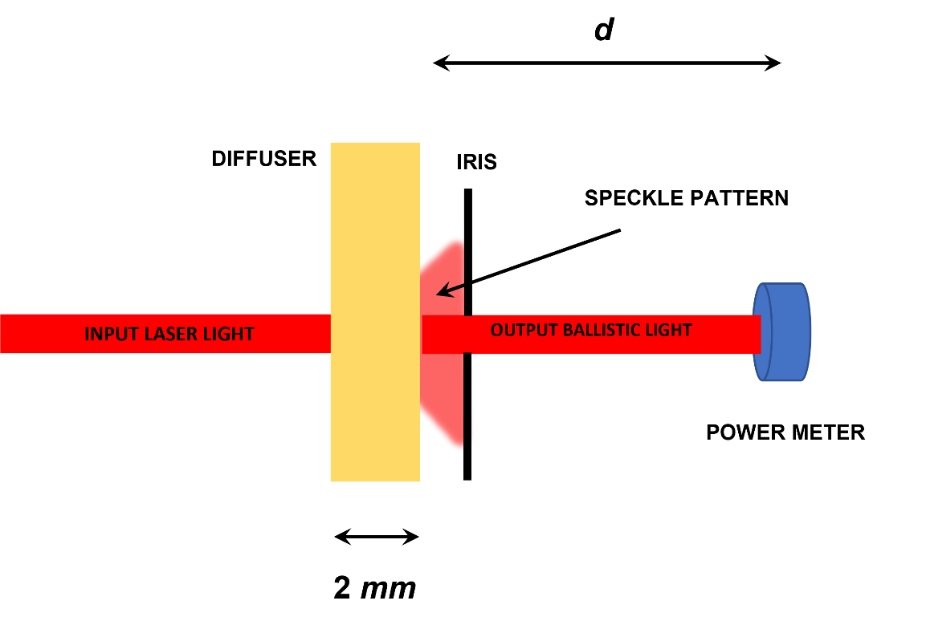


Figure S1 The experimental setup to measure the optical depth and the mean free path.

First, the value of the input intensity ($I_{0}$) is measured by the power meter without the diffuser. The output intensity (I) from the diffuser is then measured by placing the diffuser of width 2 mm in the optical path of the laser. The distance between the power meter and the diffuser (D) is 10 cm. The background radiation is also measured by blocking the laser light and is deducted from the input intensity. The beam's spot size is 1 mm, and the radius of the tunable aperture in the iris is also 1 mm.

Using the values obtained from the experiment, the optical depth of the sample is calculated as:

$$d_{s}=\mu_{t}D=-ln\left( \frac{1.30*{10}^{-6} W}{1.6*{10}^{-3} W} \right)=7.11$$

And the optical mean free path is calculated as:

$$\Lambda=\frac{2 mm}{7.11}= 0.28 mm.$$

S.3 Refrernces

1. M. Lyu, H. Wang, G. Li, S. Zheng, and G. Situ, "Learning-based lensless imaging through optically thick scattering media," Adv. Photonics **1**, 1 (2019).

2. R. Michels, F. Foschum, and A. Kienle, "Optical properties of fat emulsions," Opt. Express **16**, 5907 (2008).

3. J. Gateau, H. Rigneault, and M. Guillon, "Complementary Speckle Patterns: Deterministic Interchange of Intrinsic Vortices and Maxima through Scattering Media," Phys. Rev. Lett. **118**, (2017).

4. D. Wang, S. K. Sahoo, X. Zhu, G. Adamo, and C. Dang, "Non-invasive super-resolution imaging through dynamic scattering media," Nat. Commun. **12**, (2021).

5. Y. Li, Y. Xue, and L. Tian, "Deep speckle correlation: A deep learning approach towards scalable imaging through scattering media," arXiv (2018).
