# Supplementary material for: Imaging through diffuse media using multi-mode vortex beams and deep learning
Source: Sci Rep. 2022 Jan 28;12:1561. doi: 10.1038/s41598-022-05358-w (PMC8799672; doi:10.1038/s41598-022-05358-w)
Supplement: Supplementary file 2 — Supplementary Information 2. [file 41598_2022_5358_MOESM2_ESM.docx]

Imaging through diffuse media using multi-mode vortex beams and deep learning: Supplemental document 2

This supplemental document tables the training and validation results obtained in the article following the removal of 32 corrupted images from the dataset (16 corrupted images from the dataset obtained using Gaussian beams and 16 corresponding images from the dataset obtained using the vortex beams).

The results of the validation for 10 training processes are shown in Table 1. It can be seen that for 6 out of 10 validation processes, the neural network achieves a better validation score when the vortex beam dataset is used. In addition, the network manages to achieve better average performance when the vortex beam dataset is used.

Table 1 Validation results of the proposed neural network for 10 training of 40 epochs each.

|  | **Imaging using Gaussian beams** | | | **Imaging using vortex beams** | | |
| --- | --- | --- | --- | --- | --- | --- |
| **Training No.** | **MSE** | **NPCC** | **PSNR (dB)** | **MSE** | **NPCC** | **PSNR (dB)** |
| 1 | 0.001947 | -0.9824 | 75.23 | 0.001838 | -0.9827 | 75.48 |
| 2 | 0.001861 | -0.9829 | 75.43 | 0.001540 | -0.9859 | 76.25 |
| 3 | 0.001845 | -0.9832 | 75.46 | 0.001955 | -0.9818 | 75.21 |
| 4 | 0.001861 | -0.9836 | 75.88 | 0.001713 | -0.9843 | 75.79 |
| 5 | 0.002306 | -0.9789 | 74.50 | 0.001692 | -0.9839 | 75.84 |
| 6 | 0.001946 | -0.9820 | 75.23 | 0.001993 | -0.9818 | 75.13 |
| 7 | 0.001925 | -0.9828 | 75.28 | 0.001748 | -0.9846 | 75.70 |
| 8 | 0.002169 | -0.9807 | 74.76 | 0.001795 | -0.9837 | 75.58 |
| 9 | 0.001843 | -0.9825 | 75.47 | 0.002172 | -0.9820 | 74.76 |
| 10 | 0.001839 | -0.9831 | 75.48 | 0.001902 | -0.9819 | 75.33 |
| **Mean** | 0.001957 | -0.9822 | 75.22 | 0.001835 | -0.9832 | 75.51 |
| **Standard deviation** | 0.000149 | 0.00133 | 0.3149 | 0.000169 | 0.00137 | 0.4005 |

The validation results show that the best result when using the data from the vortex beam dataset is -0.9859, in terms of NPCC. When the Gaussian beam dataset is used for the validation, the best result is -0.9836, in terms of NPCC. The validation results hint that the proposed neural network converges better when the vortex beam dataset is used.

Therefore, to confirm our assumptions, the neural network is tested using the weights that achieved the best results on the validation data for each dataset. Following this, a prediction was made by the network on each of the test dataset images. It is found that, when employing vortex beams for image reconstruction, the NPCC in the test phase is -0.9850. However, when using Gaussian beams for imaging acquisition, the NPCC in the training phase is -0.9837. An enhancement of 0.62 dB, in terms of PSNR, is achieved using this method when a highly scattering diffuser of grit 220 and width 2 mm (7.11 times the mean free path) is used.

To reach a firm conclusion on the results, the neural network is also tested using the weights that achieved the worst results on the validation data for each dataset. It is found that, when employing vortex beams for image reconstruction, the NPCC in the test phase is -0.9805. However, when using Gaussian beams for imaging acquisition, the NPCC in the training phase is -0.9795. An enhancement of 0.21 dB in terms of PSNR.

From the best and worst results of the testing phase using both datasets, the proposed neural network converges better when the vortex beam dataset is used.

The training results with the test data is shown in table 2.

Table 2 test dataset result.

|  | **Imaging using Gaussian beams** | | | **Imaging using vortex beams** | | |
| --- | --- | --- | --- | --- | --- | --- |
|  | **MSE** | **NPCC** | **PSNR (dB)** | **MSE** | **NPCC** | **PSNR (dB)** |
| **Best result** | 0.001861 | -0.9837 | 75.88 | 0.001640 | -0.9850 | 76.50 |
| **Worst result** | 0.002294 | -0.9795 | 75.01 | 0.002212 | -0.9805 | 75.22 |

Changes were observed when the results were obtained using corrected datasets compared to the previous results reported in the manuscript. The changes may be due to the following:

1. The removal of corrupted images from the Gaussian dataset, where one or more of the Gaussian beams might not have been captured, might have improved the neural network's performance when the Gaussian dataset is used.
2. The neural network is a powerful and deep neural network that provides accuracy irrespective of the beam used for imaging. It must be noted that the results provided in this manuscript using both the gaussian dataset and the OAM dataset clearly outperforms existing methods.

It should also be noted that the performance of the proposed neural network depends heavily on the initialization of the hyperparameters used in the training phase. Since the training batches are always selected randomly from the training data, variations are to be expected in the validation results, depending on the weights and biases acquired from the training process and the training dataset.

Moreover, different types of neural networks and datasets using the same optical method could greatly vary the results depending on the complexity of the neural network and the datasets.
